# State Racism Index and physical function: The REasons for Geographic and Racial Differences in Stroke (REGARDS) study

**DOI:** 10.1093/gerona/glag142

**Published:** 2026-06-24

**Authors:** Hugo G Quezada-Pinedo, Tyson Brown, Ene M Enogela, Emily B Levitan, Oluwasegun P Akinyelure, Valerie A Smith, Laura C Pinheiro, Monika M Safford, Ro-Jay Reid, Tomi F Akinyemiju, C Barrett Bowling

**Affiliations:** Department of Population Health Sciences, Duke University School of Medicine, Durham, North Carolina, United States; Department of Sociology and Population Research Institute, Duke University, Durham, North Carolina, United States; Department of Epidemiology, University of Alabama at Birmingham, Birmingham, Alabama, United States; Department of Epidemiology, University of Alabama at Birmingham, Birmingham, Alabama, United States; Department of Epidemiology, University of Alabama at Birmingham, Birmingham, Alabama, United States; Department of Population Health Sciences, Duke University School of Medicine, Durham, North Carolina, United States; Department of Medicine, Division of General Internal Medicine, Duke University School of Medicine, Durham, North Carolina, United States; Department of Biostatistics and Bioinformatics, Duke University School of Medicine, Durham, North Carolina, United States; Department of Medicine, Division of General Internal Medicine, Weill Cornell Medicine, New York, New York, United States; Department of Medicine, Division of General Internal Medicine, Weill Cornell Medicine, New York, New York, United States; Department of Medicine, Division of General Internal Medicine, Weill Cornell Medicine, New York, New York, United States; Department of Population Health Sciences, Duke University School of Medicine, Durham, North Carolina, United States; Duke Cancer Institute, Duke University School of Medicine, Durham, North Carolina, United States; Department of Veterans Affairs, Durham Geriatrics Research Education and Clinical Center, Durham, North Carolina, United States; Department of Medicine, Duke University, Durham, North Carolina, United States; (Medical Sciences Section)

**Keywords:** Physical capacity, Physical ability, Systemic racism, Cohort studies, United States of America

## Abstract

**Background:**

Structural racism contributes to health inequities in the United States. This study aimed to quantify the association between state racism index (SRI) and physical function.

**Methods:**

In a national United States community-based cohort study, 13 661 non-Hispanic Black and White adults who had baseline information (2003-2007), SRI data (2006-2010) and physical function data (2013-2016) were included. Physical function measurements included activities of daily living (ADL), instrumental activities of daily living (IADL), timed walk, and chair stand test. Multivariable generalized regression models (GLMs) and linear regression models were used to evaluate the association between SRI and physical function. Interactions with age, sex, and region were evaluated.

**Results:**

Among Black participants, each unit increase in SRI was significantly associated with 2% higher IADL scores (ratio of means [95% confidence interval, CI]: 1.018 [1.007, 1.029]) indicating worse function. This association attenuated after adjustment for socioeconomic factors but was stronger in the United States Stroke Belt region (*p* for interaction < .05). Among White participants, higher SRI was significantly associated with 2% ADL and 1% IADL lower scores (ratio of means [95% CI]: 0.983 (0.969, 0.997) and 0.985 (0.977, 0.994), respectively) indicating better function. This association was independent of socioeconomic and health-related factors. We did not observe an association between SRI and timed walk or chair stands overall or by race.

**Conclusion:**

Higher state-level structural racism was associated with worse physical function among Black participants and better physical function for White participants. Associations were influenced by socioeconomic factors and magnified in southern United States.

## Introduction

The concept of structural racism acknowledges that racism goes beyond individual prejudices and is perpetuated through laws, regulations, and practices at various levels of government, becoming deeply embedded in societal norms and institutional systems.^1^^,^^2^ Structural racism is recognized as a key factor contributing to inequities in education, income, and health behaviors that accumulate over the lifespan and across generations and may lead to worse downstream health outcomes including disparities in chronic disease-related mortality.^2^ To capture the complex nature of structural racism, novel and robust measures such as the state racism index (SRI), which combines indicators of residential segregation, incarceration rates, educational attainment, economic status, and employment, have been developed and shown to be associated worse health outcomes.^3^^,^^4^

The effects of structural racism might be especially important for older, Black adults. Previous hypotheses suggest that racism generates inequalities that accumulate over a person’s life and can result in poor health outcomes.^5^ An important, and understudied outcome in this population is physical function, as maintaining functional independence is often a critical goal for older adults.^6^ While the impact of individual risk factors on physical function, including race, has been extensively studied, the effects of broader social issues such as structural racism on function and independence remain relatively unexplored.^7–11^

The objective of our study was to investigate the association between SRI and a battery of self-reported functional measures and physical performance tests in participants of the REasons for Geographic And Racial Differences in Stroke (REGARDS) study. Additionally, we aimed to examine these associations stratified by race and to explore potential interaction effects of age, sex, and region. This study aims to improve our understanding of structural factors that may influence physical function and potentially enable the early identification of individuals at higher risk of impaired physical function. These insights could inform the development of population health interventions to improve physical function.

## Methods

### Study population

This study used data from the prospective, United States population-based REGARDS cohort study. REGARDS was designed to identify the causes of elevated stroke mortality among Non-Hispanic Black and residents of the Stroke Belt region.^12^ From 2003 to 2007, REGARDS enrolled 30 239 individuals from 48 states who identified as Black or White, oversampling Black individuals and residents from the high-stroke mortality Stroke Belt and Stroke Buckle regions of the Southeastern United States.^13^^,^^14^ At that time, participants had a computer-assisted telephone interview and an in-home exam. Between 2013 and 2016, participants were invited to participate in a second in-home exam.

**Figure 1:**
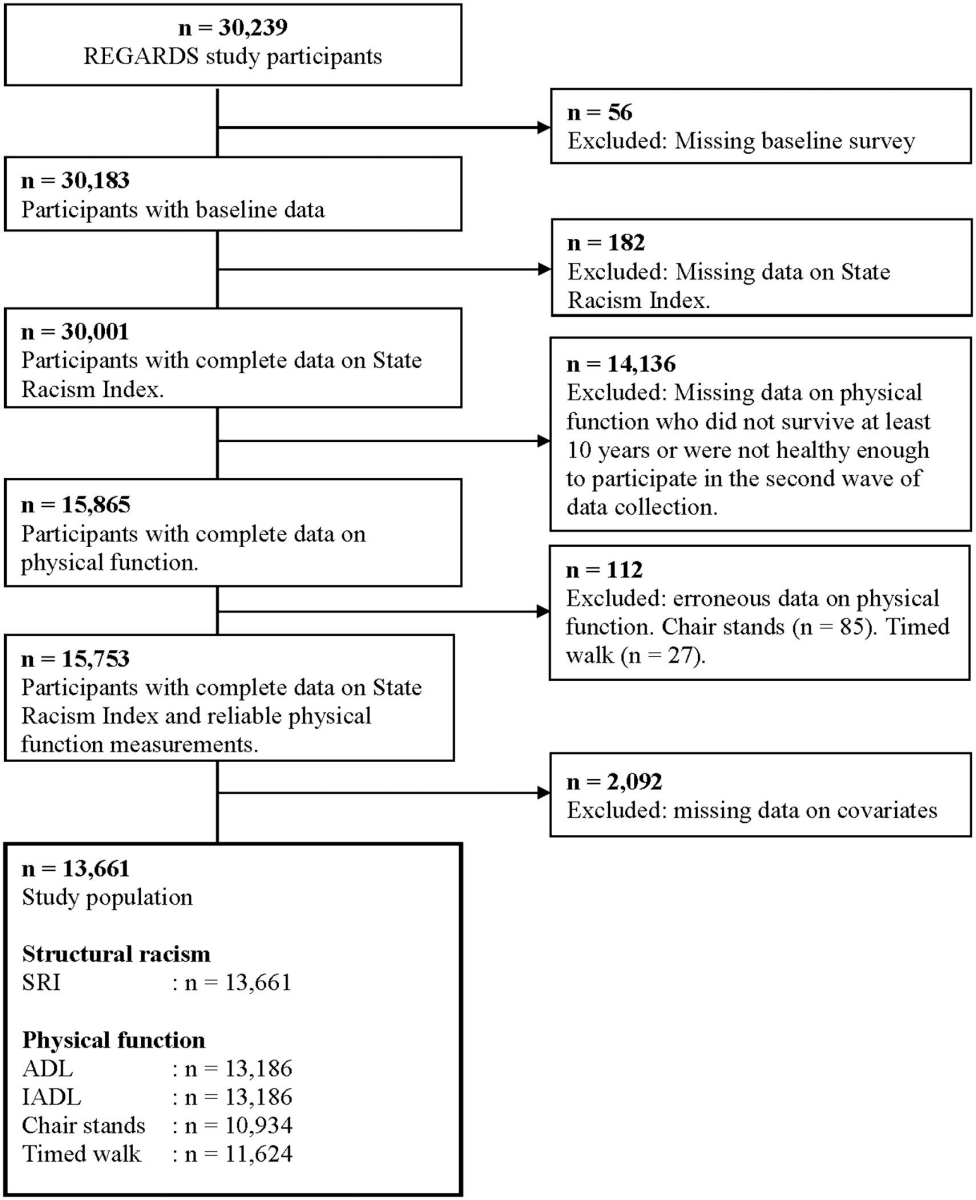
Flow chart of the participants included in the analysis.

Our analyses were restricted to 15 865 REGARDS participants who had data available for SRI from 2006 to 2010 and physical function measured at the second in-home exam in 2013-2016. As such, individuals in our sample had to survive at least 10 years to complete the second in-home visit. Participants with erroneous data on physical function (*n* = 112) and no information on covariates (*n* = 2092) were also excluded, resulting in a final analytic cohort of 13 661 participants (Figure 1).

### SRI

SRI was calculated for 2 time periods (2006-2010 and 2013-2015) to overlap as best possible with the 2 REGARDS in-home exams (baseline: 2003-2007, second in-home exam: 2013-2016) given the availability of public available data for the SRI (Figure S1, Tables S1-S3).^4^^,^^15^ Since the effects of structural racism may take time to manifest,^16^ we selected the 2006-2010 period as our primary time period for SRI. SRI was available for all states and evaluates 5 domains of structural racism: residential segregation, incarceration, education, economics, and employment where higher scores indicate higher disparities between Black and White residents of a State.^4^^,^^15^ The residential segregation domain evaluates 2 components: the index of dissimilarity, and the isolation index.^4^ The incarceration domain included the Black-White ratio of the incarceration rate.^4^ The education domain included the Black-White ratio of the sample without a college degree.^4^ The economic domain included the Black-White ratios of poverty, Black-White ratios of population that live in rental housing as opposed to owning a home, and a White-Black ratio of household income.^4^ The employment domain included the Black-White ratio of the population not participating in the labor force and the Black–White ratio of unemployment.^4^ The total SRI score was calculated by averaging the scores across the 5 domains and ranges from 0 to 100, with higher scores indicating greater structural racism.^4^ Consistent with prior studies, these results reflect spatial differences in contemporary racialized regimes, distinct configurations of racism that manifest in different ways across regions of the United States. The measure used in this study, like those employed in earlier work, captures institutional manifestations of structural racism, which are particularly salient in Midwestern and Northeastern states.^3^^,^^17–20^ By contrast, cultural forms of racism have been found to be especially pronounced in southern United States.^21^ These regional patterns illustrate how racism operates through multiple, context-dependent modalities, revealing its dynamic, flexible, and adaptive nature in shaping racialized regimes across place.

### Physical function measurements

Physical function measurements were obtained at the second in-home examination and included 2 distinct types of measures: self-reported physical functioning measurements (activities of daily living [ADL], instrumental activities of daily living [IADL]) and performance measurements (timed walk, chair stands) (Table S4).^22^ Self‑reported function measures captured participants’ perceived difficulty performing daily tasks; specifically, ADLs included the following 5 activities: getting out of bed or chair, eating, dressing, bathing, and using a toilet.^22^ IADL included the following 7 activities: household chores, purchasing items, planning and preparing meals, managing money, using a telephone, taking medications, and traveling by vehicle.^22^ All activities were categorized according to the participant response into 0 (I could do it by myself with no difficulty), 1 (I could do it by myself with some difficulty) and 2 (I would need someone to help me do it). The final ADL and IADL scores were calculated by summing all relevant activities.^23^ For the ADL score, the range was from 0 to 10, while for the IADL score, the range was from 0 to 14.^22^ In both cases, a higher score indicates worse function.^22^ In contrast, physical performance measures objectively assessed physical capability during the in-home visit; specifically, timed walk was evaluated during in-home visits as the average time of 2 completed 8-foot timed walks utilizing a cane or walker if needed.^22^ Chair stands were evaluated by measuring the time to completion of 5 chair stands which involved standing up and sitting down without using arms.^22^ Measurements above 1 minutes or below 1 second were considered erroneous and not included in this analysis (Figure 1).^22^

### Covariates

Selection of covariates was based on the WHO International Classification of Function, that is based on a biopsychosocial model that describes functional decline as a consequence of the interaction between health conditions and personal and environmental contextual factors.^22^ Thus, the following covariates at baseline were included: socioeconomic covariates (annual household income, participant education level), demographic covariates (age at baseline, sex, REGARDS region: Stroke Belt and non-Belt), percentage of the state’s population made up of Black residents, duration of residence in the state reported at baseline (years) and health-related covariates (body mass index, smoking status and history of heart disease which included self-reported myocardial infarction [MI], coronary artery bypass grafting [CABG], bypass, angioplasty, stenting or evidence of MI on ECG).

### Statistical analysis

First, descriptive statistics of participant characteristics were presented overall and categorized by SRI 2006-2010 tertiles in Table 1 to visualize potential relationships between patient characteristics and SRI levels. Second, to explore potential selection bias, we compared characteristics between study participants and those who did not participate in our analysis. Third, the association between SRI 2006-2010 and IADL and ADL scores were evaluated using generalized regression models (GLMs) with a negative binomial distribution and a log link to model count data with skewed distributions. Then, the ratios of means (95% confidence interval [CI]) were calculated as the exponentiated beta coefficients (95% CI) and interpreted as the percentage difference in the number of ADL and IADL scores. For timed walk and chair stands, linear regression was used to calculate the mean difference in time to completion (95% CI). Empirical variance sandwich estimators were used to calculate robust CIs and *p*-values in all our models. Model assumptions were evaluated by visual inspection of diagnostic plots. To prevent over adjustment, we adopted a minimal adjustment approach, carefully selecting covariates based on previous literature. In the demographic models, we adjusted for baseline age, sex, race, region and percentage of Black individuals residing in the state. In the socioeconomic model, we additionally adjusted for baseline annual household income and education. In the health-related models, we additionally adjusted for baseline body mass index, smoking status, history of heart disease and stroke. Variance inflation factors (VIFs) were used to evaluate multicollinearity among parameters in our models and no evidence of multicollinearity was identified (VIF < 10).^24^ Fourth, in the final model (health-related model), we examined whether the association between SRI and physical function outcomes varied by age (<65 vs. 65+), sex (male vs. female), and region (Stroke Belt vs. not) by separately adding interaction terms: (SRI*age), (SRI*sex), and (SRI*region). Fifth, to assess whether changes in the SRI are linked to physical function, we further examined the difference between SRI at follow-up and SRI at baseline in relation to physical function. Sixth, to determine which SRI components may play the largest part, in the full model, we evaluated each indicator individually. Seventh, to account for the potential influence of non-normally distributed timed walk and chair stands outcomes on our results, we applied a generalized linear model with a Gaussian distribution and a log link. Eighth, to evaluate whether duration of residence in the state reported at baseline influenced the results, we additionally adjusted our fully adjusted models for the duration of residency in that state. All statistical analyses were performed in R software version 4.4.0 (R Foundation for Statistical Computing, Vienna, Austria).

**Table 1:** Participant’s characteristics by SRI 2006-2010 tertile.

	**Total (*n* = 13** **661)**	SRI tertile 1 (*n* = 289)	SRI tertile 2 (*n* = 6085)	SRI tertile 3 (*n* = 4687)	*p*-value T1 versus T2	*p*-value T2 versus T3
**Demographic**						
Age categories, *n* (%)						
<56	2495 (18.3)	546 (18.9)	1100 (18.1)	849 (18.1)	.358	.171
56-59	2529 (18.5)	555 (19.2)	1076 (17.7)	898 (19.2)		
60-65	3571 (26.1)	741 (25.6)	1567 (25.8)	1263 (26.9)		
66-69	2014 (14.7)	405 (14.0)	933 (15.3)	676 (14.4)		
70-75	1961 (14.4)	419 (14.5)	895 (14.7)	647 (13.8)		
76-79	688 (5.0)	140 (4.8)	323 (5.3)	225 (4.8)		
80-85	365 (2.7)	73 (2.5)	175 (2.9)	117 (2.5)		
≥86	38 (0.3)	10 (0.3)	16 (0.3)	12 (0.3)		
Sex, *n* (%)						
Female	7536 (55.2)	1530 (53.0)	3522 (57.9)	2484 (53.0)	<.001	<.001
Male	6125 (44.8)	1359 (47.0)	2563 (42.1)	2203 (47.0)		
Race, *n* (%)						
Black	5107 (37.4)	1179 (40.8)	2149 (35.3)	1779 (38.0)	<.001	.005
Whites	8554 (62.6)	1710 (59.2)	3936 (64.7)	2908 (62.0)		
Region population, *n* (%)						
Stroke Belt	7589 (55.6)	1486 (51.4)	4355 (71.6)	1748 (37.3)	<.001	<.001
Non-belt	6072 (44.4)	1403 (48.6)	1730 (28.4)	2939 (62.7)		
**Socioeconomic**						
Income, *n* (%)						
<$20 000	1998 (14.6)	381 (13.2)	850 (14.0)	767 (16.4)	.203	<.001
$20 000-$34 000	3434 (25.1)	691 (23.9)	1501 (24.7)	1242 (26.5)		
$35 000-$74 000	5127 (37.5)	1151 (39.8)	2283 (37.5)	1693 (36.1)		
≥$75 000	3102 (22.7)	666 (23.1)	1451 (23.8)	985 (21.0)		
Education, *n* (%)						
Less than high school	1039 (7.6)	198 (6.9)	433 (7.1)	408 (8.7)		
High school graduate	3189 (23.3)	642 (22.2)	1335 (21.9)	1212 (25.9)		
Some college	3691 (27.0)	806 (27.9)	1638 (26.9)	1247 (26.6)		
College graduate and above	5742 (42.0)	1243 (43.0)	2679 (44.0)	1820 (38.8)	.705	<.001
**Health related factors**						
Body mass index, kg/m²	29.4 (6.0)	29.4 (5.9)	29.3 (6.0)	29.6 (6.2)	.312	.018
Smoking, *n* (%)						
Never	6598 (48.3)	1411 (48.8)	2980 (49.0)	2207 (47.1)		
Past	5461 (40.0)	1155 (40.0)	2429 (39.9)	1877 (40.0)		
Current	1602 (11.7)	323 (11.2)	676 (11.1)	603 (12.9)	.991	.012
History of heart disease, yes, *n* (%)	1830 (13.4)	383 (13.3)	763 (12.5)	684 (14.6)	.358	.002
Stroke, yes, *n* (%)	510 (3.7)	105.0 (3.6)	216.0 (3.6)	189.0 (4.0)	.883	.210
**Structural racism**						
SRI 2006-2010, mean (SD)	43.7 (5.0)	39.3 (1.0)	41.6 (0.8)	49.0 (5.1)	<.001	<.001
SRI 2013-2015, mean (SD)	47.5 (8.4)	43.1 (4.9)	46.8 (7.0)	51.0 (10.0)	<.001	<.001
**Physical function**						
Self-reported measurements:						
ADL scores, mean (SD)	0.3 (1.0)	0.2 (0.9)	0.3 (1.0)	0.3 (1.0)	.017	.757
IADL scores, mean (SD)	1.1 (2.3)	1.1 (2.2)	1.1 (2.3)	1.2 (2.3)	.166	.724
Observed measurements:						
Chair stands, seconds, mean (SD)	14.1 (6.4)	14.1 (6.5)	14.1 (6.2)	14.1 (6.5)	.777	.663
Timed walk, seconds, mean (SD)	6.1 (5.5)	7.0 (6.2)	5.6 (4.9)	6.3 (5.7)	<.001	<.001

Abbreviations: ADL, activities of daily living; IADL, instrumental activities of daily living; SRI: state racism index (higher SRI indicates greater structural racism). SRI 2006-2010 range: 37-68. SRI 2013-2015 range: 26-75. SRI Q1: ≤40. SRI Q2: >40 and ≤ 43. SRI Q3: >43. Stroke Belt region: North Carolina, South Carolina, Georgia, Tennessee, Mississippi, Alabama, Louisiana, and Arkansas.

## Results

### Participant characteristics

**Figure 2:**
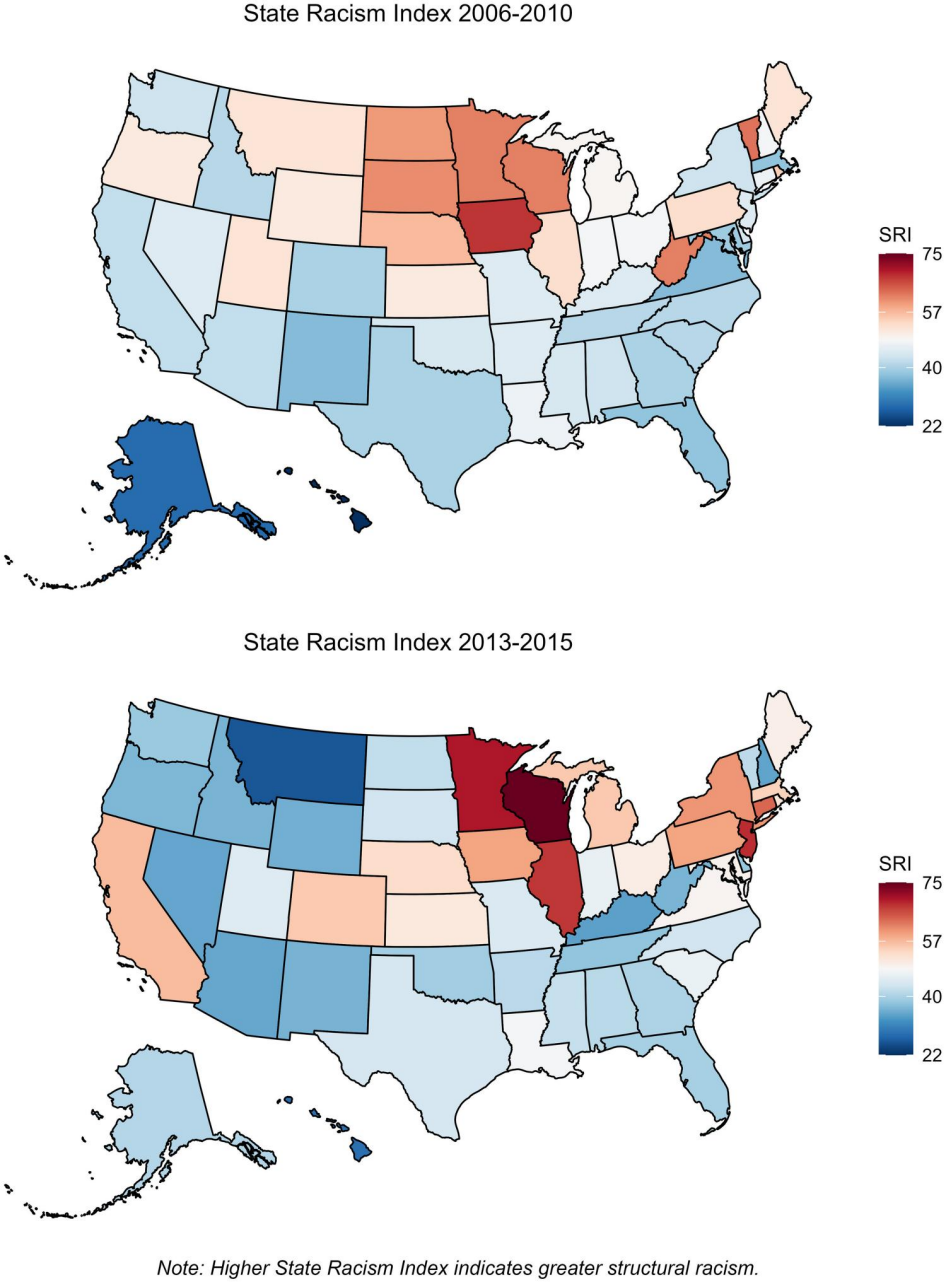
Distribution of state racism index in United States. Higher state racism index indicates greater structural racism.

Participants’ characteristics, stratified by SRI tertiles, are summarized in Table 1. The mean for SRI was 43.7 (SD = 5.0), for ADL was 0.3 (SD = 1.0), for IADL was 1.1 (SD = 2.3), for chair stands was 14.1 seconds (SD = 6.4), and for timed walk was 6.1 seconds (SD = 5.5). Additionally, 62.9% of the participants were 65 years or younger, and 55.2% were female. In 2006-2010, higher SRI scores were observed in the MidWest region with a notable increase in the SRI score in these regions in 2013-2015 (Figure 2). Included participants were younger, more likely to be White, nonsmoker, less likely from the Stroke Belt region, less likely to have stroke, and had higher income, higher education levels, higher BMI compared to participants who were excluded and less likely to have history of heart disease (*p*-values < .05) (Table S5).

### SRI and physical function

The associations of SRI with physical function overall, and stratified by race, are presented in Table 2. In the overall study population, no association between SRI and physical function measurements was observed. Likewise, in the race stratified analysis, SRI was not associated with any performance measures. In contrast, for the self-reported function measures, among Black participants, in the unadjusted models, 1 unit increase in SRI was associated with higher IADL scores (ratio of means [95% CI]: 1.018 [1.007, 1.029]) indicating lower physical function. After adjustment for demographic characteristics, higher SRI was associated with higher ADL and IADL scores (ratio of means [95% CI]: 1.025 [1.001, 1.050] and 1.021 [1.008, 1.035], respectively). No other statistically significant associations between SRI and other physical function measurements were observed among Black participants. Among White participants, in our unadjusted models, 1-unit increase in SRI was associated with lower ADL and IADL scores (ratio of means [95% CI]: 0.983 [0.969, 0.997] and ratio of means [95% CI]: 0.985 [0.977, 0.994], respectively). After adjustment for demographic and socioeconomic characteristics, 1 unit increase in SRI was associated with lower IADL scores (ratio of means [95% CI]: 0.987 [0.977, 0.996]). After additional adjustment for health-related characteristics, higher SRI was associated with lower IADL scores (ratio of means [95% CI]: 0.988 [0.979, 0.998]). No other associations for SRI and physical function were observed among White participants.

**Table 2:** Association of state racism index and physical function by race.

	Self-reported measurements		Observed measurements	
Baseline state racism index	Activities of daily living ratio of means (95% CI)	Instrumental activities of daily living ratio of means (95% CI)	Timed walk, mean difference (95% CI)	Chair stands, mean difference (95% CI)
**Total population**				
Unadjusted model	0.997 (0.986, 1.008)	0.997 (0.991, 1.004)	−0.006 (−0.025, 0.013)	−0.013 (−0.037, 0.011)
Demographic model	1.004 (0.991, 1.017)	1.001 (0.994, 1.009)	0 (−0.02, 0.021)	0.008 (−0.018, 0.034)
Socioeconomic model	0.995 (0.982, 1.008)	0.993 (0.986, 1.001)	−0.008 (−0.029, 0.012)	−0.007 (−0.032, 0.019)
Health-related model	0.998 (0.984, 1.011)	0.995 (0.987, 1.002)	−0.009 (−0.030, 0.012)	−0.009 (−0.034, 0.018)
**Black participants**				
Unadjusted model	1.017 (0.997, 1.038)	1.018 (1.007, 1.029) *	0.019 (−0.019, 0.057)	0.008 (−0.041, 0.058)
Demographic model	1.025 (1.001, 1.050) *	1.021 (1.008, 1.035) *	0.035 (−0.003, 0.073)	0.037 (−0.015, 0.088)
Socioeconomic model	1.010 (0.988, 1.034)	1.009 (0.997, 1.023)	0.013 (−0.025, 0.051)	0.008 (−0.044, 0.059)
Health-related model	1.008 (0.984, 1.033)	1.008 (0.995, 1.022)	0.012 (−0.026, 0.05)	0.002 (−0.049, 0.053)
**White participants**				
Unadjusted model	0.983 (0.969, 0.997) *	0.985 (0.977, 0.994) *	−0.013 (−0.035, 0.009)	−0.016 (−0.044, 0.011)
Demographic model	0.992 (0.976, 1.007)	0.992 (0.983, 1.001)	−0.015 (−0.04, 0.009)	−0.006 (−0.037, 0.024)
Socioeconomic model	0.987 (0.971, 1.004)	0.987 (0.977, 0.996) *	−0.020 (−0.045, 0.005)	−0.013 (−0.043, 0.017)
Health-related model	0.990 (0.974, 1.006)	0.988 (0.979, 0.998) *	−0.019 (−0.044, 0.006)	−0.013 (−0.043, 0.017)

Values for activities of daily living and instrumental activities of daily living represent ratio of means (95% confidence intervals [CIs]) calculated using generalized regression model with a negative binomial distribution and a log link. These values reflect the ratio of the mean number of physical activity scores per unit increase in baseline state racism index (where higher state racism index indicates greater structural racism). Values for timed walk and chair stands represent linear regression coefficients (95% CI). These values reflect the change in physical activity measurements per unit increase in state racism index. These values reflect the change in physical activity measurements per unit increase in baseline state racism index. Demographic models were adjusted for age, sex, race (for total population only), region and percentage of Black population in the state. Socioeconomic models were additionally adjusted for income and education. Health-related models were additionally adjusted for body mass index, smoking, cardiovascular disease history and stroke. Bold values represent *p*-value < .05.

* *p* < .05.

Among Black participants only, an interaction between SRI and age in relation to ADL, and between SRI and region in relation to all physical activity measures were identified (*p* for interaction < .05) (Table 3). In the analysis by region, among participants living in the Belt region, higher SRI was associated with higher ADL and IADL scores, timed walk and chair stands (ratio of means [95% CI]: 1.108 [1.035, 1.188], ratio of means [95% CI]: 1.078 [1.031, 1.127], β [95% CI]: 0.122 [0.017, 0.227] seconds and β [95% CI]: 0.146 [0.011, 0.280] seconds, respectively), indicating lower physical function. In the analysis by age, among participant with age < 65-years, higher SRI was associated with higher ADL indicating lower physical function. Among White participants, an interaction between SRI and age in relation to chair stands and between SRI and region in relation to timed walk were found (*p* for interaction < .05). However, no significant associations were observed within age and region strata.

**Table 3:** Association of state racism index and physical function by sex and age.

	Self-reported measurements		Observed measurements	
State racism index	Activities of daily living ratio of means (95% CI)	Instrumental activities of daily living ratio of means (95% CI)	Timed walk mean difference (95% CI)	Chair stands mean difference (95% CI)
**Black participants**				
**Age**				
<65 years	1.036 (1.003, 1.007) *	1.008 (0.989, 1.026)	0.017 (−0.028, 0.063)	0.022 (−0.041, 0.084)
≥65 years	0.975 (0.947, 1.005)	1.010 (0.994, 1.027)	0.008 (−0.058, 0.074)	−0.028 (−0.116, 0.061)
*p* for interaction	**.002**	.956	.388	.902
**Sex**				
Female	1.014 (0.987, 1.043)	1.009 (0.994, 1.024)	0.012 (−0.040, 0.064)	−0.037 (−0.108, 0.035)
Male	1.016 (0.972, 1.062)	1.014 (0.990, 1.038)	0.013 (−0.039, 0.065)	0.059 (−0.013, 0.131)
*p* for interaction	.486	.843	.900	.109
**Region**				
Belt	1.108 (1.035, 1.188) *	1.078 (1.031, 1.127) *	0.122 (0.017, 0.227) *	0.146 (0.011, 0.280) *
Non-Belt	0.997 (0.970, 1.024)	1.001 (0.987, 1.015)	0.043 (0.001, 0.085) *	−0.012 (−0.069, 0.044)
*p* for interaction	**.001**	**<.001**	**<.001**	**<.001**
**White participants**				
**Age**				
<65 years	0.980 (0.956, 1.006)	0.982 (0.968, 0.997) *	−**0.033 (**−**0.065,** −0.002) *	0.004 (−0.034, 0.043)
≥65 years	0.994 (0.973, 1.015)	0.991 (0.979, 1.004)	−0.009 (−0.050, 0.031)	−0.044 (−0.091, 0.002)
*p* for interaction	.574	.218	.9423	**.028**
**Sex**				
Female	0.997 (0.974, 1.021)	0.986 (0.973, 1.000) *	−0.024 (−0.058, 0.010)	−0.006 (−0.055, 0.044)
Male	0.982 (0.959, 1.006)	0.990 (0.975, 1.004)	−0.015 (−0.051, 0.021)	−0.019 (−0.055, 0.018)
*p* for interaction	.971	.595	.278	.838
**Region**				
Belt	0.992 (0.925, 1.065)	0.995 (0.957, 1.034)	0.068 (−0.015, 0.150)	−0.015 (−0.124, 0.094)
Non-Belt	0.993 (0.974, 1.012)	0.988 (0.976, 0.999) *	−0.006 (−0.035, 0.024)	−0.022 (−0.055, 0.012)
*p* for interaction	.327	.198	**<.001**	.716

Values for activities of daily living and instrumental activities of daily living represent ratio of means (95% confidence intervals [CIs]) calculated using generalized regression model with a negative binomial distribution and a log link. These values reflect the ratio of the mean number of physical activity scores per unit increase in baseline state racism index (where higher state racism index indicates greater structural racism). Values for timed walk and chair stands represent linear regression coefficients (95% CI). These values reflect the change in physical activity measurements per unit increase in state racism index. Models were be adjusted for age, sex (except for the sex stratified analysis), race (except for the race stratified analysis), region (except for the region stratified analysis) and percentage of Black population in the state, income and education, body mass index, smoking, cardiovascular disease history and stroke. Stroke Belt region: North Carolina, South Carolina, Georgia, Tennessee, Mississippi, Alabama, Louisiana, and Arkansas. Bold values represent *p*-value < .05.

* *p*-value < .05.

### Supplemental analyses

Associations between SRI and physical function by sex and age and region for the overall study population are presented in Table S6. There was an interaction effect between SRI and region in relation to timed walk and chair stands (*p* for interaction < .05). In the Belt region, higher SRI was associated with higher IADL scores and timed walk (ratio of means [95% CI]: 1.030 [1.001, 1.1062] and β [95% CI]: 0.092 [0.027, 0.157] seconds, respectively), indicating lower physical function. In the non-Belt region, higher SRI was associated with lower IADL (ratio of means [95% CI]: 0.991 [0.983, 1.000]), indicating better physical function.

When exploring components of SRI, multiples associations were observed with no consistent trend in their directions (Table S7). Among Black participants, segregation index was associated with timed walk (β [95% CI]: −0.166 [−0.261, −0.072] seconds), incarceration index was associated with timed walk (β [95% CI]: −0.018 [−0.03, −0.005] seconds) and educational attainment index was associated with IADL (ratio of means [95% CI]: 1.007 [1.001, 1.013]) and timed walk (β [95% CI]: 0.039 [0.019, 0.059] seconds). For White participants, segregation index was associated with chair stands (β [95% CI]: 0.104 [0.018, 0.190] seconds), incarceration index was associated with timed walk (β [95% CI]: −0.016 [−0.026, −0.007] seconds), educational attainment index was associated with timed walk (β [95% CI]: 0.024 [0.010, 0.037] seconds), economic status index was associated with ADL (ratio of means [95% CI]: 0.990 [0.981, 1.000]) and IADL (ratio of means [95% CI]: 0.994 [0.990, 1.000]) and employment index was associated with timed walk (β [95% CI]: −0.017 [−0.035, −0.000] seconds). Among the total population, segregation index was associated with chair stand, incarceration index was associated with IADL and timed walk while educational attainment index was associated with timed walk. No other associations were observed for other SRI components and physical function measurements.

When evaluating the change of SRI between 2006-2010 and 2013-2015, a higher SRI change between 2006-2010 and 2013-2015 was associated with lower timed walk in all participants (β [95% CI]: −0.024 [−0.039, −0.008] seconds). Among Black participants, a higher SRI change between 2006-2010 and 2013-2015 was associated with lower timed walk (β [95% CI]: −0.054 [−0.085, −0.024] seconds) and lower chair stands (β [95% CI]: −0.051 [−0.093, −0.008] seconds) (Table S8). No other associations were observed between changes in the SRI from 2006-2010 to 2013-2015 and physical function measures.

Additional adjustments to account for the non-normal distribution of timed walk and chair stands outcomes showed results with similar effect sizes and directions (Tables S9 and S10). Further adjustment for residence duration did not change our conclusions showing similarity in the effect sizes and the direction of effects (Tables S11 and S12).

## Discussion

In this prospective population-based cohort study of Black and White US adults, higher levels of structural racism measured at the state level using a novel, comprehensive and multi-domain index was associated with self-reported physical function. Specifically, higher SRI was associated with worse self-reported physical function among Black participants and better self-reported physical function for White participants. These associations were likely influenced by socioeconomic factors and possibly magnified in southern United States (ie, Stroke Belt region). Furthermore, no associations between SRI and physical function measured by physical performance tests were found. These findings suggest that assessing broader social issues like structural racism through a novel and multi-domain measure can offer valuable insights into individual risk for adverse physical function.

Although the magnitude of associations between SRI and self-reported function measures was small and no association between SRI and physical performance measures was found, these findings could be meaningful at the population level. Previous studies have suggested that even small shifts in scores can have a profound impact on the number of cases with disability at the population level.^25^ Our study identified an association between SRI and self-reported function measures, but not with physical performance measures. This discrepancy likely reflects the distinct aspects of physical function captured by each approach. Self-reported function measures capture physical function abilities in real-world contexts and how people carry out their daily social roles and activities, rather than any specific physical movements such as walking or standing.^26^ In contrast, physical performance measures focus on specific, context-isolated physical movements such walking, standing that are performed under structured testing conditions.^26^ Because self-reported function measures reflect one’s function under real-world conditions, they may be more sensitive to environmental contextual factors, such as neighborhood safety and walkability, and access to public transportation, factors which may be influenced by SRI. It is therefore possible that the impact of SRI is more likely to be experienced in daily activities than in brief physical tasks. Another possibility is that declines in physical performance may have already occurred earlier in the participant’s life, reducing variability at the time of assessment and limiting the ability to detect associations with SRI. Self-reported functioning, in theory, could be shaped by expectations or by differential reporting across groups. However, the REGARDS study lacks data on functional expectations that it would be necessary to further explore this possibility.

Findings from this study provide evidence that structural racism may negatively influence physical function. A previous county level study found that structural racism was associated with lower well-being and that this negative effect was stronger in counties where Black population was higher.^27^ Moreover, the way in which structural racism operates appears to benefit White than Black participants as our study found that higher SRI was associated with better IADL scores among White participants. This aligns with previous evidence showing no effect or beneficial effects at higher levels of structural racism on various health outcomes among White population.^28^^,^^29^

When examining our results by geographic region, we identified stronger associations across all physical function measurements in southern United States defined as the Stroke Belt region. The interaction between race and geography may be particularly relevant, especially for Black participants residing in the Belt region, an area that has historically experienced a disproportionate burden of cardiovascular diseases and longstanding structural inequities.^30^ Among Black individuals living in the Belt region, the association between SRI and physical function was consistent across both self-reported function and physical performance measures and demonstrated the largest effect sizes. These findings suggest that the influence of structural racism may vary depending on both race and geographic location. This study extends the existing literature by highlighting potential factors that may help explain the disproportionate burden of chronic disease in the Belt region. Further studies are needed in smaller geographic areas within this region to better understand the associations observed in our study. In our supplementary analysis, which examined each individual component of the SRI, we identified ambiguous results with no clear directional trend. Notably, results from some components of SRI were contrary to our hypothesis (e.g., higher inequities in economic status were correlated with lower inequities in education attainment, and higher segregation index was associated with better timed walk in the Black participants). Although the reasons for these findings are unclear, we cautiously speculate that adaptive processes operating at sub‑state levels may be influencing these patterns. Prior work suggests that neighborhood characteristics can foster stronger social connections among neighbors, enhance perceived social control, and reduce uncertainty regarding different social norms.^31^ For example, residing in a neighborhood with a shared racial identity may promote social cohesion and support, ultimately contributing to better health outcomes.^31^

These ambiguous results could be also attributed to the limited capacity of the individual SRI components as unidomain measures, which may not effectively capture the complex interrelationships between the components of structural racism. Thus, SRI may provide a greater predictive value as a comprehensive, multicomponent measure of structural racism compared to individual measures alone. The SRI maps illustrate important state level patterns of structural racism that are different than individual level factors because they reflect broader contextual forces. Given the differences in effect directions observed in the component analyses of SRI, future work could examine how structural racism measured at more granular levels may shape individual functional outcomes. In an additional evaluation of changes in SRI from 2006-2010 to 2013-2015, we found inconsistent results such as an increase in SRI was associated with lower timed walk in the total population and lower timed walk and chair stands among Black participants. This inconsistency may reflect a latency effect, particularly for the 2013-2015 period, as the influence of the structural racism exposure on physical function outcome may require time to manifest potentially limiting our abilities to detect associations and leading to bias in our estimates.^16^ Moreover, the diminished capacity to recover physical function as people age^32^ and differential survival rates among Black participants with worse function might have obscured the effects of subsequent exposure to higher levels of SRI. Additionally, in the age stratified analyses, we observed significant associations only among participants under 65 years of age. This pattern may indicate that the greater burden of functional limitations among older adults makes differences between exposed and unexposed groups more difficult to detect.

Notably, physical function may serve as an indicator of structural racism, reflecting disparities in resource distribution and access.^33^ Structural racism can affect physical function through individual factors such as obesity and cardiac disease and environmental factors such as lack of transportation and public spaces.^28^^,^^29^^,^^33^^,^^34^ Structural racism can also influence other aspects of daily life, influencing household crowding, housing quality, and accessibility, thereby restricting activities within the home environment.^33^ It can also influence employment and earnings, reducing flexibility for a healthy work-life balance and limiting opportunities for leisure-time physical activity.^33^ Structural racism can also affect credit access, constraining residential choices and perpetuating disparities in housing quality.^33^ Parkinson’s disease can also affect physical function however, the prevalence reported in the REGARS cohort is lower than <1%^35^ for which we believe it would not have a relevant contribution to our results.

Our sensitivity analyses that additionally adjusted for the non-normal distribution of the timed walk and chair stand outcomes and for the duration of residency were largely consistent with the primary analyses, suggesting that our identified associations were robust.

The study strengths were its large sample size and its population-based prospective design with detailed data on physical function and socio-demographic and health related factors. Our study focuses on SRI, which is recognized as an influential social stressor.^15^ Using a longitudinal epidemiological methodology, we considered in the analysis baseline covariates and left out variables at follow up that are more likely to be mediators, as their inclusion might lead to biased results.^36^ Timing of SRI evaluation is crucial, as the effects of structural racism on health might be gradual, and accumulate over the life course through cumulative exposure to socioeconomic disadvantages, limited access to resources, and chronic stress.^2^ Thus, evaluation of SRI before physical function is essential for more accurate evaluation of our associations. This study utilized multiple measures of physical function, offering a comprehensive assessment of overall physical performance. When comparing characteristic between the study participants and those lost to follow-up, participants exhibited better health and higher socioeconomic status than non-participants. These differences are expected as participants need to survive long enough and remain in the study to complete the second assessment. Selection toward a wealthier and healthier population could influence our findings if the observed associations differ between those included and excluded from the analysis, potentially limiting our ability to identify associations. Assessing racism at the state level may not fully capture its impact at other subnational geographic scales. However, previous research has demonstrated that the effect sizes of structural racism remain consistent across both state and local levels.^28^ Whether SRI follows a similar pattern, however, remains uncertain. The variability of SRI in the Belt regions was relatively low (range 40-47). Nevertheless, we were able to identify consistent associations across all physical function measures.

## Conclusions

In a large, United States community-based study, higher structural racism measured at the state-level using a novel multi-domain index was associated with worse physical function among Black participants and better physical function for White participants. These associations were likely influenced by socioeconomic factors and possibly magnified in southern United States. Evaluation of broader issues like structural racism using novel, multi-domain measures may enhance our understanding of individual risk for impaired physical function.

## Supplementary Material

glag142_Supplementary_Data

## Data Availability

REGARDS data are not publicly available due to ethical and legal restrictions. To abide by its obligations with NIH/NINDS and the Institutional Review Board of the University of Alabama at Birmingham, REGARDS facilitates data sharing through data use agreements. Any investigator is welcome to access the REGARDS data, including statistical code, through this process. Requests for data access may be sent to regardsadmin@uab.edu.
