# Supplementary material for: State Racism Index and physical function: The REasons for Geographic and Racial Differences in Stroke (REGARDS) study
Source: J Gerontol A Biol Sci Med Sci. 2026 Jun 24;81(7):glag142. doi: 10.1093/gerona/glag142 (PMC13318489; doi:10.1093/gerona/glag142)
Supplement: glag142_Supplementary_Data [file glag142_supplementary_data.zip › Supplement_Data.zip!/20290527_SRI_PF_supp.docx]

**Supplemental material**

**State Racism Index and physical function: The REasons for Geographic and Racial Differences in Stroke (REGARDS) Study**

Hugo G Quezada-Pinedo MD, PhD, Tyson Brown, PhD, Ene M. Enogela, MPH, Emily B Levitan, ScD, Oluwasegun P Akinyelure, MD, PhD, Valerie A. Smith, DrPH, Laura C. Pinheiro, MPH, PhD, Monika M. Safford, MD, MPI, Ro-Jay Reid, MD, MSc, Tomi F. Akinyemiju, PhD, C Barrett Bowling, MD, MSPH

Contents

[Supplementary Methods	3](#_Toc226553752)

[Supplementary figure S1.	4](#_Toc226553753)

[Supplementary Table S1. Definitions, data sources, and missing data for state racism measures	5](#_Toc226553754)

[Supplementary Table S2 State racism measures, 2006-2010	6](#_Toc226553755)

[Supplementary Table S3. State racism measures, 2013-2015	8](#_Toc226553756)

[Supplementary Table S4. Physical function measurements	10](#_Toc226553757)

[Supplementary Table S5. Participant’s characteristics for those included and not included.	11](#_Toc226553758)

[Supplementary Table S6. Association of State Racism Index and physical function by sex and age and region (Total population).	12](#_Toc226553759)

[Supplementary Table S7. Association of State Racism Index components and physical function by race.	13](#_Toc226553760)

[Supplementary Table S8. Association of State Racism Index change and physical function by race.	14](#_Toc226553761)

[Supplementary Table S9. Association of State Racism Index and physical function by race, accounting for non‑normal outcome distributions.	15](#_Toc226553762)

[Supplementary Table S10. Association of State Racism Index and physical function by sex and age, accounting for non‑normal outcome distributions.	16](#_Toc226553763)

[Supplementary Table S11. Association of State Racism Index and physical function by race, accounting for how long participants resided in the baseline state.	17](#_Toc226553764)

[Supplementary Table S12. Association of State Racism Index and physical function by sex and age, accounting for how long participants resided in the baseline state.	18](#_Toc226553765)

Supplementary Methods

**State Racism Index**

SRI was calculated for the 2006-2010 and 2013-2015 periods to overlap as best possible with REGARDS baseline (2003-2007) and 2^nd^ in-home exam (2013-2016) given the availability of public available data in the SRI (Supplementary methods, Figure S1, Table S1-3).^1,2^ This structural racism: residential segregation, incarceration, education, economics, and employment where higher scores indicate higher disparities between Black and White residents of a state.^1^ The residential segregation dimension evaluates two components: the index of dissimilarity, which measures the differential residential distribution of two racial groups, and the isolation index, which measure the spatial isolation of one racial group from another. The index of dissimilarity was calculated with the formula: $D=\frac{1}{2}sum\left( \mathrm{blackpct}-\mathrm{whitepct} \right)*100$.^1^ Where “*blackpct”* is the proportion of the state’s Black population living in each block and *“whitepct”* is the proportion of the state’s White population living in that block.^1^ The isolation index was calculated with the formula: $I = 100-(SUM ((blackpct)*(proportionblack)) * 100)$.^1^ Where “*blackpct”* is the proportion of the state’s Black population living in each block and “*proportionblack”* is the proportion of people in that block who are Black.^1^ The incarceration dimension included the incarceration rate gap calculated as Black population incarceration rate to White population incarceration rate for each state considering prisoners under the jurisdiction of state or federal correctional authorities regardless of where the prisoner is held.^1^ The education dimension included education attainment calculated as the ratio of proportion of Black population without a college degree to the proportion of White population without a college degree for each state.^1^ The economic dimension consisted of three components; the poverty status gap ratio as the ratio of proportion of Black population living under the poverty level to the proportion of White population living under the poverty level for each state, the median annual household income gap calculated as the ratio of White population median annual household income to Black population median annual household income for each state, and the rental housing percent gap calculated as the ratio of proportion of Black population in rental housing to proportion of White population in rental housing for each state.^1^ The employment dimension consisted of two components; the non-labor force participation calculated as the ratio of proportion of Back population not participating in the labor force to proportion of White population not participating in the labor force for each state, and the unemployment as ratio of proportion of unemployed Black population to proportion of unemployed White population for each state.^1^ The dissimilarity index and isolation index were calculated in a 0-100 scale.^1^ For the other indices, the ratios were normalized to a 0-100 scale.^1^ The total SRI score was calculated by averaging the scores across the five dimensions, with values from 0 to 100, with higher scores indicating greater structural racism.^1^ SRI was lowest in Alaska (22) and highest in Iowa (68) in 2006-2010, while in 2013-2015, it was lowest in Montana (26) and highest in Wisconsin (75) (Supplementary Table S2-3).

Supplementary Figure S1.

Supplementary Table S1. Definitions, data sources, and missing data for state racism measures

| **Dimension** | **Measure** | **Description** | **Data source** |
| --- | --- | --- | --- |
| Segregation | Dissimilarity Index | D = 1/2 SUM [blackpct - whitepct] * 100, where blackpct is the proportion of the state’s Black population living in each block and whitepct is the proportion of the state’s White population living in that block. Values are on a scale from 0-100 with 100 being the most spacially segregated by race. It represents the percentage of Black population that would have to move in order to achieve an equal distribution of White and Black population across all blocks within a state. | U.S. Census Data |
|  | Isolation Index | I = 100 - (SUM((blackpct)*(proportionblack)) * 100), where blackpct is the proportion of the state’s Black population living in each block and proportionblack is the proportion of people in that block who are Black. Values are on a scale from 0-100 with higher values representing higher levels of segregation. It can be interpreted as the probability a Black person does not share a block area with a White person or as the extent to which Black members of a block are exposed only to one another. | U.S. Census Data |
| Incarceration | Incarceration Rate Gap | Ratio of Black population incarceration rate to White population incarceration rate for each state. | U.S. Bureau of Justice Statistics and US Census Data |
| Education | Educational Attainment Gap | Ratio of proportion of Black population without a college degree to the proportion of White population without a college degree for each state. | U.S. Census Data |
| Economic | Poverty Status Gap Ratio | Ratio of proportion of Black population living under the poverty level to the proportion of White population living under the poverty level for each state. | U.S. Census Data |
|  | Median Annual Household Income Gap | Ratio of White population median annual household income to Black population median annual household income for each state. | U.S. Census Data |
|  | Rental Housing Percent Gap | Ratio of proportion of Black population in rental housing to proportion of White population in rental housing for each state. | U.S. Census Data |
| Employment | Non-Labor Force Participation | Ratio of proportion of Black population not participating in the labor force to proportion of White population not participating in the labor force for each state. | U.S. Census Data |
|  | Unemployment | Ratio of proportion of unemployed Black population to proportion of unemployed White population for each state. | U.S. Census Data |

Source: Mesic et al., 2018.^1^

Supplementary Table S2 State racism measures, 2006-2010

| **State** | **Segregation index** | **Incarceration index** | **Educational attainment index** | **Economic status index** | **Employment index** | **State Racism Index** |
| --- | --- | --- | --- | --- | --- | --- |
| Alabama | 51 | 9 | 46 | 57 | 52 | 43 |
| Alaska | 70 | 10 | 30 | 14 | 17 | 28 |
| Arizona | 66 | 18 | 66 | 21 | 41 | 42 |
| Arkansas | 59 | 17 | 56 | 44 | 50 | 45 |
| California | 67 | 28 | 43 | 26 | 47 | 42 |
| Colorado | 72 | 31 | 10 | 30 | 60 | 40 |
| Connecticut | 65 | 71 | 0 | 53 | 48 | 47 |
| Delaware | 52 | 27 | 43 | 51 | 46 | 44 |
| Florida | 56 | 19 | 40 | 36 | 38 | 38 |
| Georgia | 50 | 6 | 40 | 59 | 47 | 40 |
| Hawaii | 70 | 0 | 20 | 9 | 9 | 22 |
| Idaho | 67 | 24 | 57 | 29 | 29 | 41 |
| Illinois | 55 | 56 | 28 | 57 | 68 | 53 |
| Indiana | 62 | 28 | 53 | 38 | 57 | 48 |
| Iowa | 71 | 85 | 51 | 67 | 66 | 68 |
| Kansas | 65 | 50 | 34 | 41 | 64 | 51 |
| Kentucky | 63 | 27 | 62 | 32 | 41 | 45 |
| Louisiana | 49 | 16 | 41 | 73 | 57 | 47 |
| Maine | 72 | 39 | 53 | 49 | 50 | 52 |
| Maryland | 52 | 23 | 19 | 56 | 47 | 39 |
| Massachusetts | 65 | 26 | 8 | 41 | 47 | 38 |
| Michigan | 56 | 35 | 43 | 50 | 60 | 49 |
| Minnesota | 69 | 76 | 29 | 71 | 65 | 62 |
| Mississippi | 46 | 0 | 46 | 75 | 55 | 44 |
| Missouri | 58 | 24 | 43 | 40 | 57 | 45 |
| Montana | 68 | 67 | 81 | 15 | 30 | 52 |
| Nebraska | 67 | 52 | 34 | 54 | 80 | 57 |
| Nevada | 63 | 16 | 65 | 34 | 46 | 45 |
| New Hampshire | 70 | 45 | 61 | 34 | 33 | 48 |
| New Jersey | 59 | 53 | 19 | 48 | 46 | 45 |
| New Mexico | 65 | 5 | 72 | 11 | 34 | 37 |
| New York | 61 | 42 | 19 | 41 | 50 | 43 |
| North Carolina | 55 | 22 | 34 | 47 | 47 | 41 |
| North Dakota | 72 | 46 | 77 | 46 | 59 | 60 |
| Ohio | 59 | 32 | 43 | 49 | 57 | 48 |
| Oklahoma | 63 | 20 | 58 | 34 | 44 | 44 |
| Oregon | 71 | 38 | 65 | 35 | 45 | 51 |
| Pennsylvania | 59 | 64 | 34 | 45 | 60 | 53 |
| Rhode Island | 71 | 63 | 27 | 60 | 51 | 55 |
| South Carolina | 50 | 20 | 26 | 60 | 50 | 41 |
| South Dakota | 73 | 71 | 53 | 29 | 80 | 61 |
| Tennessee | 56 | 16 | 54 | 35 | 44 | 41 |
| Texas | 61 | 10 | 52 | 30 | 47 | 40 |
| Utah | 68 | 45 | 61 | 33 | 54 | 52 |
| Vermont | 68 | 82 | 100 | 17 | 46 | 63 |
| Virginia | 54 | 29 | 9 | 46 | 50 | 37 |
| Washington | 70 | 33 | 38 | 31 | 40 | 43 |
| West Virginia | 71 | 100 | 72 | 20 | 49 | 62 |
| Wisconsin | 62 | 77 | 34 | 60 | 77 | 62 |
| Wyoming | 70 | 33 | 59 | 44 | 47 | 51 |

Source: Alvarez et al., 2023^2^ Note: Higher State Racism Index indicates greater structural racism.

Supplementary Table S3. State racism measures, 2013-2015

| **State** | **Segregation index** | **Incarceration**  **index** | **Educational index** | **Economic**  **status**  **index** | **Employment**  **index** | **State Racism Index** |
| --- | --- | --- | --- | --- | --- | --- |
| Alabama | 71 | 17 | 42 | 34 | 42 | 41 |
| Alaska | 41 | 14 | 69 | 36 | 45 | 41 |
| Arizona | 44 | 26 | 48 | 28 | 27 | 35 |
| Arkansas | 71 | 22 | 37 | 33 | 43 | 41 |
| California | 60 | 65 | 84 | 28 | 48 | 57 |
| Colorado | 52 | 47 | 100 | 30 | 49 | 56 |
| Connecticut | 63 | 71 | 96 | 51 | 39 | 64 |
| Delaware | 59 | 27 | 44 | 32 | 31 | 39 |
| Florida | 65 | 22 | 56 | 27 | 29 | 40 |
| Georgia | 69 | 14 | 49 | 30 | 39 | 40 |
| Hawaii | 37 | 0 | 90 | 1 | 14 | 29 |
| Idaho | 45 | 19 | 15 | 30 | 72 | 36 |
| Illinois | 76 | 70 | 70 | 54 | 68 | 68 |
| Indiana | 67 | 34 | 38 | 43 | 50 | 46 |
| Iowa | 52 | 78 | 46 | 63 | 56 | 59 |
| Kansas | 54 | 49 | 62 | 38 | 53 | 51 |
| Kentucky | 57 | 21 | 31 | 30 | 32 | 34 |
| Louisiana | 73 | 23 | 49 | 45 | 51 | 48 |
| Maine | 52 | 37 | 26 | 74 | 61 | 50 |
| Maryland | 73 | 34 | 76 | 27 | 39 | 50 |
| Massachusetts | 58 | 43 | 93 | 40 | 39 | 55 |
| Michigan | 73 | 50 | 49 | 50 | 57 | 56 |
| Minnesota | 57 | 82 | 69 | 87 | 55 | 70 |
| Mississippi | 74 | 12 | 41 | 40 | 46 | 42 |
| Missouri | 71 | 23 | 48 | 34 | 46 | 45 |
| Montana | 46 | 29 | 9 | 34 | 11 | 26 |
| Nebraska | 60 | 62 | 43 | 51 | 52 | 53 |
| Nevada | 46 | 13 | 48 | 29 | 37 | 35 |
| New Hampshire | 41 | 20 | 42 | 32 | 37 | 34 |
| New Jersey | 70 | 96 | 86 | 48 | 44 | 69 |
| New Mexico | 43 | 32 | 50 | 23 | 33 | 36 |
| New York | 78 | 62 | 81 | 30 | 50 | 60 |
| North Carolina | 63 | 25 | 56 | 31 | 42 | 43 |
| North Dakota | 47 | 31 | 35 | 66 | 30 | 42 |
| Ohio | 67 | 39 | 47 | 48 | 52 | 50 |
| Oklahoma | 57 | 26 | 37 | 34 | 42 | 39 |
| Oregon | 43 | 33 | 37 | 34 | 36 | 37 |
| Pennsylvania | 71 | 68 | 56 | 43 | 58 | 59 |
| Rhode Island | 54 | 69 | 65 | 38 | 33 | 52 |
| South Carolina | 66 | 27 | 64 | 34 | 42 | 47 |
| South Dakota | 51 | 39 | 28 | 61 | 37 | 43 |
| Tennessee | 70 | 21 | 37 | 27 | 35 | 38 |
| Texas | 64 | 22 | 63 | 32 | 40 | 44 |
| Utah | 40 | 49 | 50 | 47 | 40 | 45 |
| Vermont | 41 | 63 | 13 | 31 | 60 | 42 |
| Virginia | 60 | 33 | 80 | 30 | 43 | 49 |
| Washington | 46 | 31 | 57 | 29 | 30 | 39 |
| West Virginia | 53 | 37 | 21 | 25 | 45 | 36 |
| Wisconsin | 73 | 98 | 65 | 64 | 75 | 75 |
| Wyoming | 46 | 23 | 46 | 31 | 32 | 36 |

Source: Mesic et al 2018.^1^ Higher State Racism Index indicates greater structural racism.

Supplementary Table S4. Physical function measurements

| Physical function measurements | Definition |
| --- | --- |
| Activities of daily living (ADL) | ADL were getting out of bed or chair, eating, dressing, bathing and using a toilet. For each activity, participants selected from the options ‘I could do it by myself with no difficulty’ (assigned a value of 0), ‘I could do it by myself with some difficulty’ (assigned a value of 1) and ‘I would need someone to help me do it’ (assigned a value of 2). We created ADL scores by summing all activities. |
| Instrumental activities of daily living (IADL) | IADL were household chores, purchasing items, planning and preparing meals, managing money, using a telephone, taking medications and travelling by vehicle  We created IADL scores by summing all activities. |
| Timed walk | During the in-home visit, participants completed two 8-foot timed walks, using canes or walkers if needed, and the results were averaged. Timed walk was estimated in seconds. Timed walk >1 min or <1 s was considered erroneous and were not included in analyses. |
| Chair stands | Participants were timed completing five chair stands (standing up and sitting down without using arms). Times >1 min or <1 s were considered erroneous and were not included in analyses. Chair stands was estimated in seconds. |

Source: Levitan et al., 2023

Supplementary Table S5. Participant’s characteristics for those included and not included.

|  | **Participant**  **(n = 13,661)** | **Non-participant**  **(n = 16,522)** | **p-value** |
| --- | --- | --- | --- |
| **Demographic** |  |  |  |
| Age categories, n (%) |  |  | <0.001 |
| <56 | 2495.0 (18.3) | 2242.0 (13.6) |  |
| 56–59 | 2529.0 (18.5) | 2185.0 (13.2) |  |
| 60–65 | 3571.0 (26.1) | 3455.0 (20.9) |  |
| 66–69 | 2014.0 (14.7) | 2314.0 (14.0) |  |
| 70–75 | 1961.0 (14.4) | 2968.0 (18.0) |  |
| 76–79 | 688.0 (5.0) | 1622.0 (9.8) |  |
| 80–85 | 365.0 (2.7) | 1327.0 (8.0) |  |
| ≥86 | 38.0 (0.3) | 409.0 (2.5) |  |
| Sex, n (%) |  |  | 0.857 |
| Female | 7536.0 (55.2) | 9096.0 (55.1) |  |
| Male | 6125.0 (44.8) | 7426.0 (44.9) |  |
| Race, n (%) |  |  | <0.001 |
| Black | 5107.0 (37.4) | 7407.0 (44.8) |  |
| White | 8554.0 (62.6) | 9115.0 (55.2) |  |
| Region population, n (%) |  |  | 0.002 |
| Non-Belt | 6072.0 (44.4) | 7357.0 (44.5) |  |
| Stroke Belt | 4621.0 (33.8) | 5826.0 (35.3) |  |
| **Socioeconomic** |  |  |  |
| Income, n (%) |  |  | <0.001 |
| <$20 000 | 1998.0 (14.6) | 3480.0 (27.2) |  |
| $20 000–$34 000 | 3434.0 (25.1) | 3873.0 (30.3) |  |
| $35 000–$74 000 | 5127.0 (37.5) | 3787.0 (29.6) |  |
| ≥$75,000 | 3102.0 (22.7) | 1652.0 (12.9) |  |
| Education, n (%) |  |  | <0.001 |
| Less than high school | 1039.0 (7.6) | 2753.0 (16.7) |  |
| High school graduate | 3189.0 (23.3) | 4615.0 (28.0) |  |
| Some college | 3691.0 (27.0) | 4399.0 (26.7) |  |
| College graduate and above | 5742.0 (42.0) | 4730.0 (28.7) |  |
| **Health related factors** |  |  |  |
| Body mass index, m/cm^2^ | 29.4 (6.0) | 29.3 (6.4) | 0.030 |
| Smoking, n (%) |  |  | <0.001 |
| Never | 6598.0 (48.3) | 7006.0 (42.7) |  |
| Past | 5461.0 (40.0) | 6606.0 (40.3) |  |
| Current | 1602.0 (11.7) | 2794.0 (17.0) |  |
| History of heart disease, yes, n (%) | 1830.0 (13.4) | 3519.0 (22.1) | <0.001 |
| Stroke, yes, n (%) | 510.0 (3.7) | 1420.0 (8.6) | <0.001 |
| **Structural racism** |  |  |  |
| SRI 2006-2010, mean (SD) | 43.7 (5.0) | 43.7 (4.9) | 0.810 |
| SRI 2013-2015, mean (SD) | 47.5 (8.4) | 47.5 (8.4) | 0.805 |

SRI: State Racism Index (higher State Racism Index indicates greater structural racism).

Supplementary Table S6. Association of State Racism Index and physical function by sex and age and region (Total population).

|  | **Self-reported measurements** | | **Observed measurements** | |
| --- | --- | --- | --- | --- |
|  | **Activities of**  **daily living**  **Ratio of means**  **(95%CI)** | **Instrumental**  **activities**  **of daily living**  **Ratio of means**  **(95%CI)** | **Timed walk**  **Beta**  **(95%CI)** | **Chair stands**  **Beta**  **(95%CI)** |
| **State Racism Index** |  |  |  |  |
|  |  |  |  |  |
| **Age** |  |  |  |  |
| <65 years | 1.003 (0.983, 1.022) | 0.989 (0.978, 1.001) | -0.017 (-0.043, 0.009) | 0.009 (-0.024, 0.042) |
| ≥65 years | 0.986 (0.969, 1.004) | 0.997 (0.987, 1.007) | -0.003 (-0.038, 0.031) | -0.038 (-0.080, 0.003) |
| P for interaction | 0.101 | 0.330 | 0.720 | 0.064 |
|  |  |  |  |  |
| **Sex** |  |  |  |  |
| Female | 1.003 (0.986, 1.021) | 0.995 (0.985, 1.004) | -0.011 (-0.039, 0.017) | -0.015 (-0.056, 0.025) |
| Male | 0.993 (0.972, 1.014) | 0.995 (0.983, 1.007) | -0.010 (-0.040, 0.021) | -0.001 (-0.034, 0.032) |
| P for interaction | 0.527 | 0.910 | 0.418 | 0.539 |
|  |  |  |  |  |
| **Region** |  |  |  |  |
| Belt | 1.047 (0.998, 1.100) | **1.030 (1.000, 1.062) *** | **0.092 (0.027, 0.157) *** | 0.043 (-0.042, 0.128) |
| Non-Belt | 0.995 (0.979, 1.010) | **0.991 (0.983, 1.000) *** | 0.008 (-0.016, 0.032) | -0.019 (-0.048, 0.010) |
| P for interaction | 0.075 | 0.0260 | **p<0.001** | **0.006** |

Values for Activities of daily living and instrumental activities of daily living represent ratio of means (95% confidence intervals) calculated using GLM with a negative binomial distribution and a log link. These values reflect the ratio of the mean number of physical activity scores per unit increase in baseline State Racism Index (higher State Racism Index indicates greater structural racism). Values for timed walk and Chair stands represent linear regression coefficients (95%CI). These values reflect the change in physical activity measurements per unit increase in State Racism Index. Models were adjusted for age, sex (except for the sex stratified analysis), race (except for the race stratified analysis), region (except for the region stratified analysis) and percentage of Black population in the state, income and education, body mass index, smoking, cardiovascular disease history and stroke. Stroke Belt region: North Carolina, South Carolina, Georgia, Tennessee, Mississippi, Alabama, Louisiana, and Arkansas. Bold values represent p-value < 0.05. * p-value < 0.05.

Supplementary Table S7. Association of State Racism Index components and physical function by race.

|  | **Self-reported measurements** | | **Observed measurements** | |
| --- | --- | --- | --- | --- |
|  | **Activities of**  **daily living**  **Ratio of means (95%CI)** | **Instrumental**  **activities**  **of daily living**  **Ratio of means (95%CI)** | **Timed walk**  **mean difference**  **(95%CI)** | **Chair stands**  **mean difference**  **(95%CI)** |
| **State Racism Index** |  |  |  |  |
|  |  |  |  |  |
| **Total population** |  |  |  |  |
| Segregation Index | 1.000 (0.965, 1.036) | 0.997 (0.978, 1.017) | -0.054 (-0.115, 0.008) | **0.102 (0.032, 0.171) *** |
| Incarceration Index | 0.998 (0.993, 1.003) | **0.997 (0.994, 1.000) *** | **-0.016 (-0.023, -0.008) **** | -0.006 (-0.015, 0.004) |
| Educational Attainment Index | 1.004 (0.998, 1.010) | 1.002 (0.998, 1.006) | **0.027 (0.016, 0.038) **** | 0.005 (-0.009, 0.018) |
| Economic Status Index | 0.996 (0.988, 1.004) | 0.997 (0.993, 1.001) | -0.008 (-0.019, 0.004) | -0.004 (-0.018, 0.011) |
| Employment Index | 1.000 (0.991, 1.009) | 0.999 (0.994, 1.004) | -0.008 (-0.023, 0.006) | -0.013 (-0.031, 0.004) |
|  |  |  |  |  |
| **Black participants** |  |  |  |  |
| Segregation Index | 1.036 (0.982, 1.092) | 1.020 (0.989, 1.052) | **-0.166 (-0.261, -0.072) *** | 0.089 (-0.003, 0.208) |
| Incarceration Index | 0.999 (0.991, 1.007) | 0.998 (0.994, 1.003) | **-0.018 (-0.03, -0.005) *** | -0.013 (-0.032, 0.005) |
| Educational Attainment Index | 1.006 (0.996, 1.016) | **1.007 (1.001, 1.013) *** | **0.039 (0.019, 0.059) **** | 0.019 (-0.007, 0.044) |
| Economic Status Index | 1.008 (0.994, 1.022) | 1.005 (0.997, 1.013) | 0.003 (-0.019, 0.026) | 0.007 (-0.025, 0.039) |
| Employment Index | 1.008 (0.993, 1.023) | 1.006 (0.998, 1.014) | 0.011 (-0.013, 0.036) | -0.006 (-0.039, 0.027) |
|  |  |  |  |  |
| **White participants** |  |  |  |  |
| Segregation Index | 0.985 (0.938, 1.033) | 0.987 (0.962, 1.013) | 0.003 (-0.077, 0.082) | **0.104 (0.018, 0.190) *** |
| Incarceration Index | 0.997 (0.990, 1.004) | 0.996 (0.992, 1.000) | **-0.016 (-0.026, -0.007) *** | -0.003 (-0.014, 0.008) |
| Educational Attainment Index | 1.004 (0.995, 1.012) | 1.000 (0.996, 1.005) | **0.024 (0.010, 0.037) *** | 0.000 (-0.016, 0.016) |
| Economic Status Index | **0.990 (0.981, 1.000) *** | **0.994 (0.990, 1.000) *** | -0.013 (-0.027, 0.001) | -0.009 (-0.026, 0.008) |
| Employment Index | 0.994 (0.983, 1.005) | 0.995 (0.989, 1.002) | **-0.017 (-0.035, -0.000) *** | -0.017 (-0.037, 0.004) |

Values for Activities of daily living and instrumental activities of daily living represent ratio of means (95% confidence intervals) calculated using GLM with a negative binomial distribution and a log link. These values reflect the ratio of the mean number of physical activity scores per unit increase in baseline State Racism Index (higher State Racism Index indicates greater structural racism). Values for timed walk and Chair stands represent linear regression coefficients (95%CI). These values reflect the change in physical activity measurements per unit increase in State Racism Index. Models were adjusted for age, sex, race, region and percentage of Black population in the state, income and education, body mass index, smoking, cardiovascular disease history and stroke. * p-value < 0.05, ** p-value < 0.001.

Supplementary Table S8. Association of State Racism Index change and physical function by race.

|  | **Self-reported measurements** | | **Observed measurements** | |
| --- | --- | --- | --- | --- |
|  | **Activities of**  **daily living**  **Ratio of means**  **(95%CI)** | **Instrumental**  **activities**  **of daily living**  **Ratio of means**  **(95%CI)** | **Timed walk**  **mean difference (95%CI)** | **Chair stands**  **mean difference (95%CI)** |
| **Total population** |  |  |  |  |
| State Racism Index change | 1.011 (1.000, 1.022) | 1.006 (1.000, 1.012) | **-0.024 (-0.039, -0.008) *** | -0.018 (-0.039, 0.003) |
|  |  |  |  |  |
| **Black participants** |  |  |  |  |
| State Racism Index change | 1.004 (0.988, 1.020) | 0.999 (0.989, 1.008) | **-0.054 (-0.085, -0.024) **** | **-0.051 (-0.093, -0.008) *** |
|  |  |  |  |  |
| **White participants** |  |  |  |  |
| State Racism Index change | 1.012 (0.996, 1.028) | 1.006 (0.999, 1.014) | -0.012 (-0.031, 0.007) | -0.007 (-0.032, 0.018) |

Values for Activities of daily living and instrumental activities of daily living represent ratio of means (95% confidence intervals) calculated using GLM with a negative binomial distribution and a log link. These values reflect the ratio of means change in physical activity scores per unit increase in State Racism Index from 2006-2010 to 2013-2015. Values for timed walk and chair stands represent linear regression coefficients (95%CI). These values reflect the change in physical activity measurements per unit increase in State Racism Index from 2006-2010 to 2013-2015. Models were adjusted for age, sex, race, region and percentage of Black population in the state, income and education, body mass index, smoking, cardiovascular disease history and stroke. * p-value < 0.05. ** p-value < 0.001.

Supplementary Table S9. Association of State Racism Index and physical function by race, accounting for non‑normal outcome distributions.

|  | **Observed measurements** | |
| --- | --- | --- |
| **Baseline State Racism Index** | **Timed walk,**  **Ratio of means**  **(95%CI)** | **Chair stands,**  **Ratio of means**  **(95%CI)** |
| **Total population** |  |  |
| Unadjusted model | 0.999 (0.996, 1.002) | 0.999 (0.997, 1.001) |
| Demographic model | 1.001 (0.997, 1.004) | 1.001 (0.999, 1.002) |
| Socioeconomic model | 0.999 (0.995, 1.002) | 0.999 (0.997, 1.001) |
| Health-related model | 0.998 (0.995, 1.002) | 0.999 (0.997, 1.001) |
| **Black participants** |  |  |
| Unadjusted model | 1.003 (0.997, 1.008) | 1.001 (0.997, 1.004) |
| Demographic model | 1.005 (0.999, 1.011) | 1.002 (0.999, 1.006) |
| Socioeconomic model | 1.002 (0.996, 1.008) | 1.000 (0.997, 1.004) |
| Health-related model | 1.001 (0.995, 1.008) | 1.000 (0.996, 1.004) |
| **White participants** |  |  |
| Unadjusted model | 0.998 (0.994, 1.002) | 0.999 (0.997, 1.001) |
| Demographic model | 0.998 (0.993, 1.002) | 0.999 (0.997, 1.002) |
| Socioeconomic model | 0.997 (0.992, 1.001) | 0.999 (0.997, 1.001) |
| Health-related model | 0.997 (0.992, 1.001) | 0.999 (0.997, 1.001) |

Values for timed walk and chair stands represent ratio of means (95% confidence intervals) calculated using GLM with a Gaussian distribution and a log link to account for non-normal distribution in the outcomes. These values reflect the ratio of the mean number of physical activity scores per unit increase in baseline State Racism Index (where higher State Racism Index indicates greater structural racism). Demographic models were adjusted for age, sex, race (for total population only), region and percentage of Black population in the state. Socioeconomic models were additionally adjusted for income and education. Health-related models were additionally adjusted for body mass index, smoking, cardiovascular disease history and stroke. Bold values represent p-value < 0.05. * p-value < 0.05.

Supplementary Table S10. Association of State Racism Index and physical function by sex and age, accounting for non‑normal outcome distributions.

|  | **Observed measurements** | | | |
| --- | --- | --- | --- | --- |
| **State Racism Index** | **Timed walk**  **mean difference**  **(95%CI)** | **Chair stands**  **Ratio of means**  **(95%CI)** | **Timed walk**  **Ratio of means**  **(95%CI)** | **Chair stands**  **Ratio of means**  **(95%CI)** |
|  | **Black participants** | | **White participants** | |
| **Age** |  |  |  |  |
| <65 years | 1.002 (0.994, 1.01) | 1.001 (0.997, 1.006) | 0.994 (0.988, 1.000) | 1.000 (0.997, 1.003) |
| ≥65 years | 1.000 (0.991, 1.01) | 0.998 (0.992, 1.004) | 0.998 (0.992, 1.004) | 0.997 (0.993, 1.000) |
| P for interaction | **0.031** | 0.293 | 0.428 | 0.078 |
| **Sex** |  |  |  |  |
| Female | 1.002 (0.993, 1.010) | 0.997 (0.992, 1.002) | 0.995 (0.990, 1.001) | 0.999 (0.996, 1.003) |
| Male | 1.001 (0.992, 1.009) | 1.004 (0.999, 1.009) | 0.998 (0.991, 1.004) | 0.999 (0.996, 1.002) |
| P for interaction | 0.621 | 0.614 | 0.220 | 0.5343 |
| **Region** |  |  |  |  |
| Belt | **1.020 (1.000, 1.041) *** | **1.010 (1.001, 1.020) *** | 1.013 (0.998, 1.029) | 0.999 (0.991, 1.007) |
| Non-Belt | 1.006 (0.999, 1.013) | 0.999 (0.995, 1.003) | 0.999 (0.994, 1.004) | 0.998 (0.996, 1.001) |
| P for interaction | **p<0.001** | **p<0.001** | **p<0.001** | 0.5838 |

Values for timed walk and chair stands represent ratio of means (95% confidence intervals) calculated using GLM with a Gaussian distribution and a log link. These values reflect the ratio of the mean number of physical activity scores per unit increase in baseline State Racism Index (where higher State Racism Index indicates greater structural racism). Models were be adjusted for age, sex (except for the sex stratified analysis), race (except for the race stratified analysis), region (except for the region stratified analysis) and percentage of Black population in the state, income and education, body mass index, smoking, cardiovascular disease history and stroke. Stroke Belt region: North Carolina, South Carolina, Georgia, Tennessee, Mississippi, Alabama, Louisiana, and Arkansas. Bold values represent p-value < 0.05. * p-value < 0.05.

Supplementary Table S11. Association of State Racism Index and physical function by race, accounting for how long participants resided in the baseline state.

|  | **Self-reported measurements** | | **Observed measurements** | |
| --- | --- | --- | --- | --- |
| **Baseline State Racism Index** | **Activities of**  **daily living**  **Ratio of means**  **(95%CI)** | **Instrumental**  **activities**  **of daily living**  **Ratio of means**  **(95%CI)** | **Timed walk,**  **mean difference**  **(95%CI)** | **Chair stands,**  **mean difference**  **(95%CI)** |
| **Total population** |  |  |  |  |
| Unadjusted model | 1.001 (0.989, 1.013) | 0.999 (0.992, 1.006) | 0 (-0.021, 0.020) | -0.013 (-0.038, 0.011) |
| Demographic model | 1.004 (0.989, 1.019) | 1.001 (0.992, 1.009) | -0.005 (-0.027, 0.018) | -0.004 (-0.032, 0.023) |
| Socioeconomic model | 0.994 (0.979, 1.009) | 0.993 (0.985, 1.001) | -0.011 (-0.033, 0.012) | -0.014 (-0.042, 0.013) |
| Health-related model | 0.997 (0.982, 1.012) | 0.994 (0.986, 1.003) | -0.011 (-0.033, 0.012) | -0.014 (-0.042, 0.013) |
| **Black participants** |  |  |  |  |
| Unadjusted model | **1.027 (1.005, 1.049) *** | **1.022 (1.01, 1.035) *** | 0.019 (-0.021, 0.06) | 0.011 (-0.044, 0.065) |
| Demographic model | **1.037 (1.009, 1.066) *** | **1.024 (1.009, 1.04) *** | 0.025 (-0.017, 0.066) | 0.035 (-0.023, 0.093) |
| Socioeconomic model | 1.016 (0.990, 1.043) | 1.009 (0.995, 1.024) | 0.003 (-0.038, 0.045) | 0.009 (-0.049, 0.067) |
| Health-related model | 1.019 (0.992, 1.048) | 1.010 (0.995, 1.026) | 0.003 (-0.038, 0.044) | 0.005 (-0.052, 0.062) |
| **White participants** |  |  |  |  |
| Unadjusted model | **0.983 (0.969, 0.998) *** | **0.987 (0.977, 0.996) *** | -0.005 (-0.028, 0.019) | -0.018 (-0.045, 0.01) |
| Demographic model | 0.987 (0.969, 1.004) | 0.991 (0.981, 1.001) | -0.017 (-0.044, 0.01) | -0.02 (-0.051, 0.012) |
| Socioeconomic model | 0.983 (0.966, 1.001) | **0.987 (0.976, 0.997) *** | -0.019 (-0.046, 0.008) | -0.023 (-0.054, 0.009) |
| Health-related model | 0.985 (0.969, 1.003) | **0.988 (0.978, 0.998) *** | -0.018 (-0.045, 0.009) | -0.022 (-0.053, 0.01) |

Values for Activities of daily living and instrumental activities of daily living represent ratio of means (95% confidence intervals) calculated using GLM with a negative binomial distribution and a log link. These values reflect the ratio of the mean number of physical activity scores per unit increase in baseline State Racism Index (where higher State Racism Index indicates greater structural racism). Values for timed walk and chair stands represent linear regression coefficients (95%CI). These values reflect the change in physical activity measurements per unit increase in State Racism Index. These values reflect the change in physical activity measurements per unit increase in baseline State Racism Index. Demographic models were adjusted for age, sex, race (for total population only), region and percentage of Black population in the state. Socioeconomic models were additionally adjusted for income and education. Health-related models were additionally adjusted for body mass index, smoking, cardiovascular disease history and stroke. Bold values represent p-value < 0.05. * p-value < 0.05.

Supplementary Table S12. Association of State Racism Index and physical function by sex and age, accounting for how long participants resided in the baseline state.

|  | **Self-reported measurements** | | **Observed measurements** | |
| --- | --- | --- | --- | --- |
| **State Racism Index** | **Activities of**  **daily living**  **Ratio of means**  **(95%CI)** | **Instrumental activities of daily living**  **Ratio of means**  **(95%CI)** | **Timed walk**  **mean difference (95%CI)** | **Chair stands**  **mean difference**  **(95%CI)** |
|  |  |  |  |  |
| **Black participants** |  |  |  |  |
| **Age** |  |  |  |  |
| <65 years | **1.047 (1.008, 1.088) *** | 1.009 (0.988, 1.031) | 0.024 (-0.028, 0.076) | 0.034 (-0.037, 0.105) |
| ≥65 years | 0.977 (0.947, 1.008) | 1.008 (0.989, 1.028) | -0.030 (-0.097, 0.038) | -0.049 (-0.149, 0.050) |
| P for interaction | **0.012** | 0.933 | **0.031** | 0.293 |
| **Sex** |  |  |  |  |
| Female | 1.019 (0.987, 1.052) | 1.009 (0.992, 1.026) | -0.005 (-0.059, 0.05) | -0.011 (-0.089, 0.067) |
| Male | 1.035 (0.984, 1.088) | 1.022 (0.993, 1.052) | 0.022 (-0.038, 0.082) | 0.030 (-0.054, 0.114) |
| P for interaction | 0.626 | 0.917 | 0.621 | 0.614 |
| **Region** |  |  |  |  |
| Belt | **1.162 (1.073, 1.257) *** | **1.091 (1.031, 1.155) *** | **0.174 (0.054, 0.293) *** | 0.136 (-0.010, 0.283) |
| Non-Belt | 1.002 (0.971, 1.034) | 1.00 (0.984, 1.016) | 0.025 (-0.020, 0.070) | -0.002 (-0.066, 0.062) |
| P for interaction | **p<0.001** | **p<0.001** | **p<0.001** | **p<0.001** |
|  |  |  |  |  |
| **White participants** |  |  |  |  |
| **Age** |  |  |  |  |
| <65 years | **0.972 (0.946, 0.998) *** | 0.986 (0.97, 1.001) | **-0.037 (-0.071, -0.003) *** | -0.005 (-0.043, 0.034) |
| ≥65 years | 0.989 (0.967, 1.013) | **0.986 (0.972, 0.999) *** | -0.004 (-0.048, 0.039) | **-0.057 (-0.109, -0.005) *** |
| P for interaction | 0.760 | 0.477 | 0.428 | 0.078 |
| **Sex** |  |  |  |  |
| Female | 0.995 (0.969, 1.021) | 0.988 (0.974, 1.003) | -0.027 (-0.064, 0.010) | -0.014 (-0.068, 0.041) |
| Male | **0.975 (0.952, 0.999) *** | 0.988 (0.973, 1.003) | -0.010 (-0.049, 0.029) | -0.027 (-0.063, 0.008) |
| P for interaction | 0.970 | 0.813 | 0.220 | 0.534 |
| **Region** |  |  |  |  |
| Belt | 0.971 (0.908, 1.038) | 0.995 (0.956, 1.036) | 0.060 (-0.024, 0.143) | 0.016 (-0.103, 0.135) |
| Non-Belt | 0.991 (0.969, 1.012) | 0.989 (0.977, 1.002) | -0.006 (-0.038, 0.026) | **-0.037 (-0.073, -0.001) *** |
| P for interaction | 0.256 | 0.136 | **p<0.001** | 0.584 |

Values for Activities of daily living and instrumental activities of daily living represent ratio of means (95% confidence intervals) calculated using GLM with a negative binomial distribution and a log link. These values reflect the ratio of the mean number of physical activity scores per unit increase in baseline State Racism Index (where higher State Racism Index indicates greater structural racism). Values for timed walk and Chair stands represent linear regression coefficients (95%CI). These values reflect the change in physical activity measurements per unit increase in State Racism Index. Models were be adjusted for age, sex (except for the sex stratified analysis), race (except for the race stratified analysis), region (except for the region stratified analysis) and percentage of Black population in the state, income and education, body mass index, smoking, cardiovascular disease history and stroke. Stroke Belt region: North Carolina, South Carolina, Georgia, Tennessee, Mississippi, Alabama, Louisiana, and Arkansas. Bold values represent p-value < 0.05. * p-value < 0.05.

**References**

1.	Mesic A, Franklin L, Cansever A, et al. The Relationship Between Structural Racism and Black-White Disparities in Fatal Police Shootings at the State Level. *Journal of the National Medical Association*. 2018/04/01/ 2018;110(2):106–116. doi:<https://doi.org/10.1016/j.jnma.2017.12.002>

2.	Alvarez CH. Structural Racism as an Environmental Justice Issue: A Multilevel Analysis of the State Racism Index and Environmental Health Risk from Air Toxics. *J Racial Ethn Health Disparities*. Feb 2023;10(1):244–258. doi:10.1007/s40615-021-01215-0
